# Midterm results after arterial switch operation for transposition of the great arteries: a single centre experience

**DOI:** 10.1186/1749-8090-7-83

**Published:** 2012-09-07

**Authors:** Aron Frederik Popov, Theodor Tirilomis, Michael Giesler, Kasim Oguz Coskun, Jose Hinz, Gerd Gunnar Hanekop, Verena Gravenhorst, Thomas Paul, Wolfgang Ruschewski

**Affiliations:** 1 Department of Thoracic and Cardiovascular Surgery, University of Göttingen, Robert-Koch-Straße, 40 37099, Göttingen, Germany; 2 Department of Anaesthesiology, Emergency and Intensive Care Medicine, University of Göttingen, Göttingen, Germany; 3 Department of Pediatric Cardiology and Pediatric Intensive Care Medicine, University of Göttingen, Göttingen, Germany; 4 Presented in part at the 8th International Conference Pediatric Mechanical Circulatory Support Systems & Pediatric Cardiopulmonary Perfusion June 13–16, 2012, Istanbul, Turkey

**Keywords:** Arterial switch operation, Transposition of great arteries, Midterm outcome

## Abstract

**Background:**

The arterial switch operation (ASO) has become the surgical approach of choice for d-transposition of the great arteries (d-TGA). There is, however an increased incidence of midterm and longterm adverse sequelae in some survivors. In order to evaluate operative risk and midterm outcome in this population, we reviewed patients who underwent ASO for TGA at our centre.

**Methods:**

In this retrospective study 52 consecutive patients with TGA who underwent ASO between 04/1991 and 12/1999 were included. To analyze the predictors for mortality and adverse events (coronary stenoses, distortion of the pulmonary arteries, dilatation of the neoaortic root, and aortic regurgitation), a multivariate analysis was performed. The follow-up time was ranged from 1–10 years (mean 5 years, cumulative 260 patient-years).

**Results:**

All over mortality rate was 15.4% and was only observed in the early postoperative period till 1994. The predictors for poor operative survival were low APGAR-score, older age at surgery, and necessity of associated surgical procedures. Late re-operations were necessary in 6 patients (13.6%) and included a pulmonary artery patch enlargement due to supravalvular stenosis (n = 3), coronary revascularisation due to coronary stenosis in a coronary anatomy type E, aortic valve replacement due to neoaortic valve regurgitation (n = 2), and patch-plasty of a pulmonary vein due to obstruction (n = 1). The dilatation of neoaortic root was not observed in the follow up.

**Conclusions:**

ASO remains the procedure of choice for TGA with acceptable early and late outcome in terms of overall survival and freedom of reoperation. Although ASO is often complex and may be associated with morbidity, most patients survived without major complications even in a small centre.

## Background

Since the introduction of the arterial switch operation (ASO) by Jatene et al. in 1975 [1], the procedure has become the surgical treatment of choice for the anatomical repair of the transposition of the great arteries (TGA). A limited number of long-term studies revealed an excellent survival and freedom from re-operation in patients undergoing ASO [2-6]. However, one of the major concerns about the ASO is the possible deterioration of neoaortic valve function with a dilatation of neoaortic root [7] and the stenoses of coronary arteries which appear occasionally and needs surgical intervention [2]. The purpose of the present study was to review our results of ASO for TGA and to evaluate early and midterm survival rates, morbidity, and complications in a single centre cohort retrospectively followed up for as long 10 years. Moreover, risk factors for mortality and adverse midterm events after ASO for TGA (coronary stenoses, distortion of the pulmonary arteries, dilatation of the neoaortic root, and aortic valve regurgitation) were the endpoints of interest.

## Methods

### Study population

The retrospective single centre study was approved by the ethics committee of the Medical Faculty, University of Göttingen. Between April 1991 and December 1999, 52 consecutive patients (median age 10 ± 49 days, range 2–338 days) underwent an ASO at the University of Göttingen.

### Data collection

All perioperative data was collected retrospectively with the help of medical records, echocardiographic and cardiac catheterization data as well operative notes. The clinical follow-up included an examination by the referring pediatric cardiologist at regular intervals or in our institution by the pediatric cardiologist. The echocardiographic data for the study was taken from the cardiologists’ report. The midterm follow-up was obtained by review of patient records and clinic notes.

### Patients’ characteristics

For 52 patients, including 37 males and 15 females, the median age at time of surgery was 10 ± 49.61 days (range, 2–338 days). The initial diagnosis was a simple TGA in 34 (66%) patients, TGA with a ventricular septal defect (VSD) in 10 (19%) patients, Taussig-Bing in 2 (4%) patients, TGA with coarctation of the aorta (CoA) in 2 (4%) patients, and TGA with CoA and VSD in 4 (8%) patients. 42 patients received Rashkind’s balloon atrial septostomy and the duration between septostomy to surgery was 7 ± 42.45 days (range, 1–271 days).

Standard surgical techniques were used. The following details were considered. A single initial infusion of crystalloid (Brettschneider solution) cardioplegia was used for the majority of the series with a dose of 30 ml/kg over 7 minutes. In five cases a total circulatory arrest with deep hypothermia (18°C) was necessary for CoA repair.

Prior to ASO, 8 patients had cardiac surgical procedures which were Blalock-Hanlon Operation (n = 3), pulmonary artery banding (n = 2), CoA correction (n = 2), and CoA correction with a an aortic arch obstruction repair (n = 1). We had 3 preterm births. One was with 31 completed weeks and with bronchopulmonary dysplasia. The second was with 35 completed weeks, and the remaining one had 36 completed weeks and had a respiratory distress syndrome and a diabetic fetopathy

A total of 34 patients (66%) were mechanically ventilated and 3 patients had a complication (intracranial hemorrhage, renal dysfunction, chest infection) before ASO. On echocardiographic examination a mean diameters of the aorta and the pulmonary artery were 11 ± 1.56 mm (range, 8-15 mm) and 12 ± 1.84 mm (range, 10-17 mm) respectively. Preoperative catheterization was performed in all patients and coronary pattern was identified with aortography or coronary angiography. It was in the beginning a standard protocol. Currently, we changed that protocol. Not every patient received a catheterization preoperative nowadays. Moreover, it was also a part of the protocol in our department to leave the chest open some cases to aid in hemodynamic and respiratory stability in the initial postoperative period.

In accordance with the Yacoub’s coronary classification [8], 45 patients were encountered for type A, 3 for type B, and another 3 for type E. One case that did not precisely fit into one of the described categories was classified as Type B1 according the Sauer classification [9]. The preoperative characteristics of the patients at the time of ASO are shown in Table 1.

**Table 1:** Preoperative characteristics

	**Patients or Mean**	**% or Range**
Age at correction (d)	10 ± 49.61	2-338
Female/Male	15/37	
Weight (g)	3505 ± 575.04	2480-5420
Body surface area (qm)	0.22 ± 0.03	0.13-0.18
***TGA pathology***		
Simple TGA	34	65.4
TGA with VSD	10	19.3
Taussig-Bing	2	3.8
TGA with CoA	2	3.8
TGA with CoA + VSD	4	7.6
***Preoperative cardiopulmonary support***		
Prostaglandin therapy	42	80.8
Duration between Rashkind maneuver to surgery	7 ± 42.45	1-271
Balloon atrial septostomy	48	92.3
***Surgery prior Switch***		
Blalock-Hanlon-Operation	3	5.7
Pulmonary artery banding	2	3.8
CoA correction	2	3.8
CoA correction and AAO repair	1	1.9
***Complications prior surgery***		
Intracranial hemorrhage	1	3.8
Renal disorder	1	3.8
Chest infection	1	3.8
Mechanical ventilatory support	34	65.4
***Vital parameter prior surgery***		
Mean arterial pressure (mmHg)	59 ± 10.49	37-85
Heart rate (beats/min)	140 ± 16.98	110-183
***Dimensions of great vessels***		
Mean diameter of the aorta (mm)	11 ± 1.56	8-15
Mean diameter of pulmonary artery (mm)	12 ± 1.84	10-17
***Coronary pattern (Yacoub)***		
A/D/E	45/3/3	86.5/5.8/5.8
***Coronary pattern (Sauer)***		
B1	1	1.9

TGA: Transposition of the great arteries; VSD: ventricular septal defect; CoA: Coarctation of the aorta;

AAO: aortic arch obstruction.

### Statistics analysis

Statistical analysis was performed by using commercial statistic software (Statistica 5.1.®, StatSoft Inc., Tulsa, OK, USA). Descriptive data for continuous variables are presented as mean ± SD, and categoric variables are presented as relatives frequencies. A multivariate analysis was performed by means of a logistic regression model for the endpoint of in-hospital mortality and by means of a Cox proportional hazard model for the endpoints of late mortality. Variables with *P* values less than 0.05 in univariate analysis were entered into the multivariate models. Freedom from time-related events was calculated using the product-limit method of Kaplan-Meier.

## Results

### Intraoperative characteristics

A standard ASO was performed in all patients, including a *Lecompte maneuver*. The techniques for the coronary artery transfer varied depending on the coronary anatomy and included the *trap door technique*.

Surgery was performed in moderate hypothermia (24°C). Myocardial protection was achieved by application of Bretschneider´s crystalloid cardioplegia (Custidiol HTK, Köhler Chemie, Germany). The right atrium was then incised and the cardioplegic solution was aspirated from coronary sinus. After transection of the great vessels, the coronary arteries were excised (in U fashion) and subsequently re-implanted in the neo-aorta. The Lecompte maneuver was performed before anastomosis of the neo-aortic root and distal ascending aorta was completed. The neo-pulmonary trunk was reconstructed with pantaloon-like untreated autologous pericardial patch. Then, the atrial septal defect (or a large foramen ovale) was closed, usually by running suture. Finally, the anastomosis between the distal pulmonary artery and the neo-pulmonary trunk was performed. Reperfusion of the heart and re-warming started after closure of the atrial septal defect.

Along with the ASO, 22 concomitant procedures were performed including closure of VSD (n = 15), closure of VSD with repair of CoA (n = 3), re-repair of CoA (n = 2), and pulmonary artery de-banding (n = 2). The mean cardiopulmonary bypass (CPB) time and the mean aortic cross clamp time was 231.5 ± 96.16 minutes (range, 129–838 minutes) and 94 ± 26.18 minutes (range, 58–152 minutes) respectively. The mean body core temperature was 24.5 ± 2.54°C (range, 17–27.5°C). In 34 (66%) patients the chest was left open for a delayed sternal closure. There were 2 (4%) intraoperative deaths which occurred during 1991–1994. All the results are summarized in Table 2.

**Table 2:** Intraoperative characteristics

	**Patients or Mean**	**% or Range**
***Additional procedures***	22	42.3
VSD closure	15	28.8
VSD closure and CoA	3	5.8
CoA re-repair	2	3.8
Debanding of pulmonary artery	2	3.8
Duration of surgery (min)	414 ± 106.56	220-865
***Intraoperative***		
CPB (min)	231.5 ± 96.16	129-838
Ischemic time (min)	94 ± 26.18	58-152
Core temperature (C°)	24.5 ± 2.54	17-27.5
Nitroprusside (μg/kg/min)	0.6 ± 0.67	0.1-3
Open chest after surgery	34	65.4
Death	2	3.8

VSD: ventricular septal defect; CoA: Coarctation of the aorta, CPB: Cardiopulmonary bypass.

### Postoperative characteristics

The postoperative characteristics of patients are shown in Table 3. Within the first 24 hours the left arterial pressure (LAP) was 12 ± 3.88 mmHg (range, 5–21 mmHg) and the mean arterial pressure was 54 ± 7 mmHg (range, 41–70 mmHg). The Levels of serum creatine phosphokinase (S-CPK) and serum creatine phosphokinase-MB (CPK-MB) were 41 ± 307 U/L (range, 41–1463 U/L) and 25.5 ± 21 U/L (range, 7–85 U/L) respectively. In intensive care unit (ICU) the transfusion of red cell and fresh frozen plasma was calculated to 294 ± 163 ml (range, 75–686 ml) and 330 ± 363 ml (range, 50–1310 ml) respectively. The duration of postoperative mechanical ventilation was 6 ± 6 days, (range, 1–28 days) and 11(25%) patients required re-intubated due to respiratory failure. The mean ICU length of stay was 10 ± 10.83 days (range, 5–57 days), and the mean hospital stay was 26 ±16.33 days (range, 14–98 days).

**Table 3:** Postperative charactistics

	**Patients or Mean**	**% or Range**
***First 24 hrs***		
LAP (mmHg)	12 ± 3.88	5-21
Mean arterial pressure (mmHg)	54 ± 7	41-70
S-CPK (U/L)	41 ± 307	41-1463
S-CPK-MB (U/L)	25.5 ± 21	7-85
***ICU***		
Red cell transfusion (ml)	294 ± 163	75-686
Fresh frozen plasma transfusion (ml)	330 ± 363	50-1310
Duration of mechanical ventilation (d)	6 ± 6	1-28
Re-intubation	11	25
Length of ICU stay (d)	10 ± 10.83	5-57
Length of hospital stay (d)	25.5 ± 16.33	14-98
***Early complications***		
Pacemaker implantation	1	2
Myocardial infarction	1	2
Rethoracotomy	1	2
***Echocardiography at discharge***		
Vmax pulmonary artery (m/s)	1.9 ± 0.63	0.9-3.2
Vmax aorta ascendens (m/s)	1.5 ± 0.29	1.2-2.9
LVEDD (mm)	21 ± 2.3	17-25.7
LVESD (mm)	14 ± 3	8-20
FS (%)	35 ± 7	22-46

LAP: left atrial pressure; S-CPK: serum creatine phosphokinase; S-CPK-MB: creatine.

Phosphokinase-MB; ICU: Intensive care unit; Vmax: peak velocity; LVEDD: left ventricular end diastolic dimension; LVESD: left ventricular end-systolic dimension: FS: Fractional shortening.

Early complication occurred in 4 patients. In 1 (2%) a pacemaker was implanted due to complete AV block, 1 (2%) had a myocardial infarction, and 1 (2%) had two re-thoracotomies. This patient had a surgical bleeding on the first postoperative day which was successfully treated and on the 22^nd^ postoperative day right atrial thrombi were detected on echocardiography, presumably due to a central venous catheter, which required surgical removal.

The postoperative echocardiography at time of discharge was performed in all patients and is summarized in Table 3. The peak velocity (Vmax) in the pulmonary artery and the aorta were 1.9 ± 0.63 m/s (range, 0.9-3.2 m/s) and 1.5 ± 0.29 m/s (range, 1.2-2.9 m/s) respectively (data diameters of the neoaortic root indexed to BSA were not available) . The fractional shortening of the left ventricle was normal in all patients (35% ± 7%; range, 22% to 46%). The measurements of LV end-diastolic and end-systolic diameters were 21 ± 2.3 mm (range, 17 to 26 mm) and 14 ± 3 mm (range, 8 to 20 mm) respectively which were within normal range.

### Outcome

All over (1991–1999) mortality rate was 15.4% (n = 8) and was observed in the early postoperative period only till 1994. Out of these patients the majority (87.5%) died in the first 30 days after ASO.

Five patients died within first 24 hours postoperatively. In two of them weaning from CPB was failed due to irreversible left heart failure and one patient had an acute refractory right heart failure due to suspected malperfusion of the right coronary artery. This patient had a type E coronary pattern according Yacoub’s classification. The remaining 2 patients underwent an uncomplicated surgery but died in ICU due to refractory left heart failure.

Two deaths occurred on second and fifth postoperative day on ICU due to multi organ failure related to a left heart failure. The last patient died on the 50^th^ postoperative day due to poor hemodynamic conditions, multiple resuscitations and the cause of death was left heart failure. That patient did not receive a control angiography, because there was so far no indication for that. The initial course went uneventful. There were no signs of myocardial infarction and the lab values did not show a myocardial ischemia. ECG and echocardiography were fine till day 20 postoperatively. Then the patient detoriated with signs of left heart failure, which resulted in a multiorgan failure (Table 4). None of our patients received a revision of the coronary arteries in the early postoperative phase.

**Table 4:** Deaths

**No.**	**Age at surgery (d)**	**Accompanied diagnosis**	**Concomitant procedure**	**Cause**
**1**	16	VSD	VSD closure	Right heart failure
**2**	41	VSD, CoA	VSD closure, CoA repair	Left heart failure
**3**	5	VSD	VSD closure	Left heart failure
**4**	20	VSD, CoA	VSD closure, CoA repair	Left heart failure
**5**	144	Taussig-Bing	-	Left heart failure
**6**	338 [preterm, 31 w]	Taussig-Bing, bronchopulmonary dysplasia	Neoaortic root enlargement with a pulmonary artery patch	Left heart failure, Multi organ failure
**7**	7 [preterm, 36 w]	-	-	Left heart failure
**8**	18 [preterm, 35 w]	respiratory distress syndrome, diabetic fetopathy	-	Left heart failure, Multi organ failure

VSD: ventricular septal defect; CoA: Coarctation of the aorta.

Late complications were observed in 7 patients and included convulsions in 1, myocardial malperfusion in 1, arrhythmia in 2, sternal wound infection in 1, and in 2 patients a chylothorax was observed.

The survivors were seen by the referring pediatric cardiologist at regular intervals or in our institution by the pediatric cardiologist. The follow-up time ranged from 1–10 years (mean 5 years, cumulative 260 patient-years) postoperatively. In majority of patients (93%), functional NYHA class I, with normal LV-function with no symptoms referable to the cardiovascular system was seen. There were no late deaths. Late re-operations were necessary in 9 (20%) patients and included a pulmonary artery patch enlargement due to supravalvular stenosis (n = 3), coronary revascularisation due to coronary stenosis in a coronary anatomy type E and aortic valve replacement due to neoaortic valve regurgitation (n = 2), and patch plasty of a pulmonary vein due to obstruction (n = 1). Two patients required pacemaker implantation due to complete AV block and one patient due to sick sinus syndrome. The dilatation of neoaortic root was not observed in any of the patients during the follow up (Table 5).

**Table 5:** Outcome

	**Patients**	**%**
***Mortality all over (1991–1999)***	8	15.4
***Late complications***		
Convulsions	1	2.2
Myocardial malperfusion	1	2.2
Arrhythmia	2	4.4
Sternal wound infection	1	2.2
Chylothorax	2	4.4
***Follow up n = 44***		
NYHA I	41	93.2
NYHA II	0	
NYHA III	3	6.8
NYHA IV	0	
Mortality	0	
***Reoperations***		
Dilatation of neoaortic root	0	
Pulmonary artery patch enlargement	3	6.6
Coronary revascularisation and aortic valve replacement	2	4.4
Patch plasty of a pulmonary vein	1	2.2
Pacemaker implantation	3	6.6

NYHA: New York Heart Association.

### Risk factors for mortality

A linear regression analysis was performed to determine possible associations of anatomic and physiologic parameters with the mortality. The predictors’ analysis for poor operative survival was: preterm birth (p = 0.0008), long duration between cardiac catheterization and surgery (p = 0.006), older age at surgery (p = 0.004), necessity of associated procedures (p = 0.004), redo surgery (p = 0.04), prolonged ischemia (p = 0.0002), and no treatment with prostaglandin prior surgery (p = 0.002) (Table 6).

**Table 6:** Risk factors for mortality

	**p-value**
Preterm birth	0.0008
Long duration between cardiac catheterization and surgery	0.006
Older age at surgery	0.004
Necessity of associated procedures	0.004
Redo surgery	0.04
Prolonged ischemia time	0.0002
No treatment with prostaglandin prior surgery	0.002

## Discussion

The goal of this study was to identify predictors for mortality and to review adverse events after ASO for TGA (coronary stenoses, distortion of the pulmonary arteries, dilatation of the neoaortic root, and aortic regurgitation). This study confirms that ASO can be performed with acceptable early and late outcome in patients with TGA even in a small centre after a learning process and modifications of the perioperative setting.

The possible deterioration of neoaortic valve function with a dilatation of neoaortic root [7] and presence of coronary stenosis [2] is one of the major concerns of ASO. In the present study, the need of surgical re-intervention of coronary stenosis in the follow up was low (4.5%). Similar results have been described by Bonhoeffer et al. for late stenoses after ASO [10]. In our patient population, 23 of the survivors who have been evaluated by angiography, 2 were found to have complete occlusion of the right coronary artery and 1 had left coronary artery occlusion. The patients with the affected right coronary artery had Yacoub’s type E coronary pattern and showed clinical symptoms, which were associated with myocardial ischemia in the year five and seven postoperatively. They were treated surgically with an internal mammary artery grafting, which is one of the possible approaches to manage coronary stenoses after ASO [11]. Although in both patients a good result were achieved with this approach, we recommend a regular follow up by angiography in view of the lack of long-term follow up studies about internal mammary grafting in children. The patient with occluded left coronary artery coronary (Yacoub’s type A coronary pattern) was treated conservatively, as the routine angiography revealed a good collateralisation and the patient had no clinical signs of a myocardial ischemia. In the latest follow up, this patient was in functional NYHA class I with no symptoms referable to the cardiovascular system.

Several investigators have noted that a deterioration of neoaortic valve function and dilatation of neoaortic root were frequently observed in patients after ASO [12,13]. In the present series two patients developed severe aortic regurgitation (AR) and required an aortic valve replacement at 6 and 8 years after ASO. One of them had a VSD which was also closed during the ASO procedure. Interestingly, both of them had Yacoub’s type E coronary pattern and received an internal mammary artery grafting as well, but there is no explanation for the combined occurrence. It was reported that the risk of moderate or severe AR with a significant left ventricular dilatation followed by an aortic valve replacement is low after ASO and the results in this study are similar to other studies [7,13]. Risk factors for AR following ASO included prior history of PA band, Taussig-Bing hearts, and development neo-aortic root enlargement [7]. Surprisingly, in our series the patients with AR who required aortic valve replacement had none of these risk factors. Moreover, we did not observe a neo-aortic root enlargement at all in the follow-up and this is in contrast to other studies which state that that is one of the major concerns of ASO [7]. This could be because of the small size of our series. Although several factors for neo-aortic root enlargement are described, these are speculative and require further investigations. The incidence of pacemaker implantation due to the complete AV block in two and the sick sinus syndrome in one was not different from that in another study [14].

The postoperative mortality and morbidity in this series was higher compared to the recently reported series. We observed an all over mortality rate of 15% (n = 8). The mortality rate seems to be high compared with other published series; however, the mortality was seen only during first 4 years of the observation period which was early nineties. Reasons for that improved outcome after 1994 could be that the ICU treatment made a progress and also the learning curve regarding the surgery has improved in a short time. Moreover, more patients were presented in a better clinical condition in the second time interval.

Seven patients died in the early postoperative period (30 days) and five of them within first 24 hrs. Cause of death was dominated mostly by postoperative refractory left heart failure. In our statistical analysis preterm birth, long duration between cardiac catheterization and surgery, older age at surgery, necessity of associated procedures, redo- surgery, prolonged ischemia, and no treatment with prostaglandin prior surgery were associated with mortality and were partly in line with other published series [6,15]. Maybe it is possible that some of the patients could be supported by an extracorporeal membrane oxygenation (ECMO) in the initial early postoperative phase; however this kind of circulatory support was first introduced in our department in the late nineties.

Interestingly, in our series the presence of a complex coronary anatomy including intramural course was not a significant risk factor for mortality and corroborates with the study of Blume et al. [16]. The results of this study show that the impact of well described risk factors influencing the mortality diminishes with surgical experience. The experience after 1994 has further improved early survival after ASO and may also result in less mid-term complications. After 1994, no postoperative deaths were observed. This result is gratifying and compares favourably with other big series [3,15].

We acknowledge that this study has several limitations being an observational assessment of outcomes. The patients were not randomly assigned to various therapies, and comparison between inherently dissimilar groups is problematic. Data regarding follow-up and re-interventions were incomplete in some patients; however continued study will be necessary for a truly long-term follow-up.

## Conclusion

In conclusion ASO remains the procedure of choice for TGA with acceptable early and late outcome in terms of overall survival and freedom of reoperation. The need for close long-term monitoring in these patients is import to detect any structural changes or changes of hemodynamic which could affect the postoperative result.

## Competing interests

There is no undisclosed ethical problem or competing interests related to the submitted manuscript.

## Authors’ contributions

AP, TT, MG participated in the design of the study and drafted the manuscript. MG, VG, KC participated in the data collection. JH participated in the statistical analysis. GH, TP and WR conceived of the study, participated in its design and coordination, helped to draft the manuscript and give final approval of the version to be published. All authors read and approved the final manuscript.

## References

[B1] Jatene AD Fontes VF Paulista PP de Souza LC Neger F Galantier M Souza JE Successful anatomic correction of transposition of the great vessels A preliminary report 1975 28 4 461 4 1200893

[B2] Kirklin J Blackstone E Tchervenkov C Castaneda A For the Congenital Heart Surgeons Society. Clinical outcomes after the arterial switch operation for transposition: patient, support, procedural and risk factors Circulation 1992 86 1501 15 10.1161/01.CIR.86.5.1501 1423964

[B3] Daebritz SH Nollert G Sachveh JS Engelhardt W Von Bernuth G Messmer BJ Anatomical risk factors for mortality and cardiac morbidity after arterial switch operation Ann Thorac Surg 2000 69 1880 86 10.1016/S0003-4975(00)01241-8 10892941

[B4] Brown JW Park HJ Turrentine MW Arterial switch operation: factors impacting survival in the current era Ann Thorac Surg 2001 71 1978 84 10.1016/S0003-4975(01)02529-2 11426778

[B5] Haas F Wottke M Poppert H Meisner H Long-term survival and functional follow-up in patients after arterial switch operation Ann Thorac Surg 1999 68 1692 97 10.1016/S0003-4975(99)01039-5 10585044

[B6] Wernovsky G Mayer JE Jonas RA Hanley FL Blackstone EH Kirklin JW Castaneda AR Factors influencing early and late outcome of the arterial switch operation for transposition of the great arteries J Thorac Cardiovasc Surg 1995 109 289 302 10.1016/S0022-5223(95)70391-8 7853882

[B7] McMahon CJ Ravekes WJ Smith EO Denfield SW Pignatelli RH Altman CA Ayres NA Risk factors for neo-aortic root enlargement and aortic regurgitation following arterial switch operation Pediatr Cardiol 2004 25 329 35 10.1007/s00246-003-0483-6 14727099

[B8] Yacoub MH Radley-Smith R Anatomy of the coronary arteries in transposition of the great arteries and methods for their transfer in anatomical correction Thorax 1978 33 4 418 24 10.1136/thx.33.4.418 694795 PMC470907

[B9] Sauer U Gittenberger-de-Groot AC Peters D Bühlmeyer K Cineangiography of the coronary arteries in transposition of the great arteries Pediatr Cardiol 1983 4 4 25 426844149

[B10] Bonhoeffer P Bonnet D Piéchaud JF Stümper O Aggoun Y Villain E Kachaner J Sidi D Coronary artery obstruction after the arterial switch operation for transposition of the great arteries in newborns J Am Coll Cardiol 1997 29 1 202 6 10.1016/S0735-1097(96)00433-0 8996315

[B11] Mavroudis C Backer CL Duffy CE Pahl E Wax DF Pediatric coronary artery bypass for Kawasaki congenital, post arterial switch, and iatrogenic lesions Ann Thorac Surg 1999 68 2 506 12 10.1016/S0003-4975(99)00588-3 10475420

[B12] Schwartz ML Gauvreau K del Nido P Mayer JE Colan SD Long-term predictors of aortic root dilation and aortic regurgitation after arterial switch operation Circulation 2004 110 128 32 10.1161/01.CIR.0000134480.06723.D8 15364851

[B13] Vandekerckhove KD Blom NA Lalezari S Koolbergen DR Rijlaarsdam ME Hazekamp MG Long-term follow-up of arterial switch operation with an emphasis on function and dimensions of left ventricle and aorta Eur J Cardiothorac Surg 2009 35 4 582 7 10.1016/j.ejcts.2008.12.034 19223194

[B14] Williams WG McCrindle BW Ashburn DA Jonas RA Mavroudis C Blackstone EH Congenital Heart Surgeon's Society. Outcomes of 829 neonates with complete transposition of the great arteries 12–17 years after repair Eur J Cardiothorac Surg 2003 24 1 1 9 10.1016/S1010-7940(03)00264-1 12853039

[B15] Dibardino DJ Allison AE Vaughn WK McKenzie ED Fraser CD Current expectations for newborns undergoing the arterial switch operation Ann Surg 2004 239 5 588 96 10.1097/01.sla.0000124293.52814.a7 15082962 PMC1356266

[B16] Blume ED Altmann K Mayer JE Colan SD Gauvreau K Geva T Evolution of risk factors influencing early mortality of the arterial switch operation J Am Coll Cardiol 1999 33 6 1702 9 10.1016/S0735-1097(99)00071-6 10334446

